# Survival and prognostic factors of anaplastic hemangiopericytoma/solitary fibrous tumor grade III

**DOI:** 10.1007/s00701-026-06843-1

**Published:** 2026-03-27

**Authors:** Alejandro Pando, Aman M. Patel, Daniel J. Valdivia, Cynthia T. Daut, Yaxel Levin-Carrion, Vraj Shah, Amar Desai, Prayag Patel, Jean Anderson Eloy, James K. Liu, Jonathan H. Sherman

**Affiliations:** 1 Department of Neurological Surgery, Rutgers New Jersey Medical School, 185 S Orange Ave, Newark, NJ 07103 USA; 2 Department of Otolaryngology – Head and Neck Surgery, Rutgers New Jersey Medical School, Newark, NJ USA; 3 Rutgers Robert Wood Johnson Medical School, New Brunswick, NJ USA; 4 Department of Internal Medicine, Rutgers New Jersey Medical School, Newark, NJ USA; 5 Department of Dermatology, Northwestern University, Chicago, IL USA; 6 Department of Neurosurgery, Cooperman Barnabas Medical Center, RWJ Barnabas Health, Livingston, NJ USA; 7 Skull Base Institute of New Jersey, Neurosurgeons of New Jersey, NYU Langone Neurosurgery Network, Livingston, NJ USA; 8 Department of Neurosurgical Oncology, Rutgers Cancer Institute of New Jersey, New Brunswick, NJ USA

**Keywords:** Anaplastic hemangiopericytoma, Solitary fibrous tumor, Malignancy, Grade III SFT/HPC

## Abstract

**Background:**

Anaplastic Hemangiopericytoma (AHPC), now known as Solitary Fibrous Tumor (SFT) Grade III, is a rare biologically aggressive neoplasm with a high rate of recurrence. Owing to its rarity, the existing literature is limited regarding its clinical characteristics, prognostic factors, management, and treatment strategies. In this study, we evaluate the association between patient demographics, clinical variables, and treatment modalities with overall survival in patients with intracranial AHPC/SFT Grade III.

**Methods:**

The National Cancer Database (NCDB) was queried for the clinical and care parameters of patients ≥ 18-years-old diagnosed with AHPC between 2004 and 2017. Multivariable Cox proportional hazards model was implemented to determine factors associated with overall survival.

**Results:**

427 patients were identified with a mean age of 52.6 ± 0.7 years. Most patients were between 40–70 years old (67.0%) with patients ≤ 40-years-old making up 21.3% and those ≥ 70-years-old making up only 11.7%. Overall median survival was 10.8 years. When stratified based on age, those ≤ 40-years-old had increased mean survival compared to those ≥ 70-years-old (10.8 vs. 6.6 years; *p* < 0.001). On multivariable analysis, increasing age (*p* = 0.006) and the receipt of chemotherapy was associated with decreased overall survival. In contrast, private insurance and managed care (*p* = 0.021), treatment with surgery alone (*p* = 0.003), or combined surgery and radiation therapy (*p* = 0.001) were independently associated with significantly improved overall survival.

**Conclusion:**

In this NCDB cohort of intracranial grade III SFT/HPC, improved overall survival was associated with younger age, private insurance status, and treatment with surgery alone or surgery combined with radiation therapy. Chemotherapy was not associated with a survival benefit. Interpretation of treatment effects should be cautious, given the potential for selection bias in chemotherapy utilization and the risk of immortal time bias analyses of radiotherapy. Continued advances in therapeutic strategies are needed to further improve survival in this rare and clinically devastating disease.

## Introduction

Anaplastic hemangiopericytoma (AHPC), reclassified as grade III Solitary Fibrous Tumor (SFT) of the central nervous system, is a rare, highly vascular and malignant tumor of mesenchymal lineage that has a poor prognosis due to its high rates of recurrence and metastasis [9, 11]. It develops from the perivascular Zimmerman pericytes (contractile spindle cells) and often involves the meninges, occasionally extending into the surrounding soft tissue [25]. Emerging evidence highlights the role of tumor microenvironment signaling and extracellular vesicle–mediated communication in driving tumor progression and immune modulation in solid tumors [16]. These neoplasms account for approximately 0.4% of all primary central  central nervous system (CNS) tumors [11]. First described by Stout and Murray [24] in 1942 and later reported in the cranial meninges by Begg and Garret [2] in 1954, these tumors were initially termed *angioblastic meningiomas* because of their radiographic and histological similarity to meningiomas. This overlap often makes them difficult to distinguish and contributes to diagnostic challenges during preoperative imaging [3, 6, 28]. 

For decades, SFTs and hemangiopericytomas (HPC) were regarded as distinct tumor entities. However, the identification of the NGFI-A-binding protein 2-signal transducer and activator of transcription 6 (NAB2-STAT6) fusion gene led to their unification into a single entity in the 2016 World Health Organization (WHO) Classification of Tumors of the Central Nervous System, which defined a combined SFT/HPC entity graded I through III [10]. Under this classification, grade I tumors are characterized by low cellularity and dense collagen deposition, corresponding to the previous diagnosis of an SFT. Grade II refers to intermediate SFT/HPC with increased cellularity and the classic “staghorn” vascular pattern. Grade III tumors corresponds to anaplastic disease (previously AHPC) and are defined by high mitotic activity (≥ 5 mitoses per 10 high-power fields) [18]. Notably, even low grade SFT/HPCs can undergo malignant transformation over time [1]. However, in the 2021 WHO classification, the term hemangiopericytoma has been retired entirely, now referring to all such tumors as SFTs with a three-tiered grading system. This change aligns CNS nomenclature with soft-tissue SFT terminology [9]. Accordingly, the historical diagnosis of AHPC currently corresponds to a grade III SFT [9]. For the purpose of this manuscript, we will refer to this entity as grade III SFT/HPC.

Early diagnosis and aggressive multimodal treatment are cornerstones in the management for grade III SFT/HPC, given the tumor’s high metastatic potential and propensity for relapse [6]. Maximal safe resection and adjuvant radiotherapy is generally recommended as first-line therapy for high-grade SFT, with the goal to improve local control and extend survival [6]. However, because SFT is an orphan disease, epidemiological studies describing clinicopathological and prognosticating factors affecting clinical progression and patient survival of an anaplastic disease are limited. To date, most published literature consists of small single-institution series and a few population-based analyses [20]. Notably, Kinslow et al. (2018) conducted the first SEER-based study under the new classification, examining outcomes across grades I-III, [7] and Kinslow et al. (2023) developed a risk stratification model for CNS SFT/HPC using national datasets [8]. Despite these advances, there remains a need for the focused analysis of the anaplastic (Grade III) subset.

This study is among the limited investigations examining patterns of care and survival specifically in patients with intracranial grade III SFTs using a large national database. We analyzed 14 years of NCDB data to identify factors associated with overall survival in this rare patient population, representing, to our knowledge, the largest multivariable analysis of outcomes in this tumor to date and contributing meaningful evidence to inform clinical management and future research.

## Methods

### Ethics

The current study being retrospective in nature, merely analyzing de-identified patient data was exempt from review by the institutional review board. It was conducted as per the principles of the International Council for Harmonization of Technical Requirements for Pharmaceuticals for Human Use (ICH) Good Clinical Practice (E6 – R2) guidelines (Maryland, USA, 2018) and in accordance with the Declaration of Helsinki (Fortaleza, Brazil, 2013).

### Study design and setting

This was a retrospective analysis of the data from the National Cancer Database (NCDB). NCDB, initiated in the year 1989, accrediting more than 1,500 health care facilities, is sponsored by both the American Cancer Society (ACS) and American College of Surgeons Commission on Cancer (CoC) [11]. The NCDB is a widely utilized and highly cited oncology registry, with extensive validation in the peer-reviewed literature supporting its reliability for large-scale outcomes research [14]. NCDB captures nearly 72% of the annual cancer diagnoses in the United States and includes records of 31 million unique patients with a new diagnosis of cancer between the years 1985 and 2015. The conclusions stated herein are, however, independent of the views of the ACS and the CoC.

### Eligibility criteria

The NCDB was queried from January 2004 through December 2017 to identify adult cases (≥ 18 years) of intracranial anaplastic hemangiopericytoma/solitary fibrous tumor. Cases were defined using International Classification of Diseases for Oncology, Third Edition, First Revision (ICD‑O‑3.1) histology and topography codes. Histology codes included 9150/3 (hemangiopericytoma, malignant) and 8815/3 (solitary fibrous tumor, malignant). Intracranial location was defined using ICD‑O‑3.1 topography codes for meninges (C70.0–C70.9) and brain (C71.0–C71.9). Patients without histologic confirmation, missing recorded vital status/survival time, or age < 18 years were excluded. NCDB histology and tumor grade variables are facility-reported and are not centrally reviewed; therefore, the designation “grade III/anaplastic” reflects the coding recorded in NCDB during the study period and may not perfectly correspond to WHO grading across all years.

### Study procedures

Anatomic site and histology were identified using ICD‑O‑3.1. Tumors were classified by ICD‑O topography as meninges (C70.0–C70.9) versus brain (C71.0–C71.9), consistent with NCDB registry coding. Patient data collected included year of diagnosis, age at diagnosis, sex, race, household income, primary payer status, facility type, Charlson-Deyo Comorbidity Index (CCI), tumor diameter, mortality, and use of primary site surgery, radiation, and chemotherapy. Relevant data were captured in a digital data extraction form and subjected to statistical analyses.

### Treatment variables

Treatment was analyzed in two complementary ways. First, surgery, radiotherapy, and chemotherapy were modeled as separate binary indicators (yes/no). Second, to improve interpretability and address collinearity among modalities, we also created mutually exclusive treatment regimen categories (e.g., no treatment; surgery only; surgery + radiotherapy; surgery + radiotherapy + chemotherapy).

### Statistical analysis plan

Demographic characteristics were summarized using descriptive statistics. The chi-squared test was implemented to compare categorical variables. The mean value of continuous variables was compared using an independent sample *t* test. Kaplan–Meier survival analysis was performed with the log-rank test to estimate mean and median survival. Overall survival (OS) was defined as time from date of diagnosis to death from any cause; patients alive at last follow-up were censored at last contact. A Cox proportional hazards regression model, including all variables from univariable Cox regression analysis with *P* < 0.05, was used for multivariable analysis. A 2-sided significance level set at *P* < 0.05 was considered statistically significant. Proportional hazards assumptions were assessed using standard diagnostic approaches (e.g., inspection of log-minus-log survival plots and testing time-by-covariate interactions). If violations were identified, stratified Cox models and/or time-varying covariate terms were considered. The regimen-based model was used to present clinically interpretable combination-treatment associations, while the modality-indicator model was used as a complementary presentation to assess modality-specific associations. Treatment variables were analyzed as first-course treatment indicators as recorded in NCDB. Because exact treatment timing relative to diagnosis and time-updated exposure modeling are not consistently available at sufficient granularity, time-dependent treatment modeling and formal landmark analyses were not performed; therefore, treatment-related hazard ratios should be interpreted as associations that may be influenced by immortal time bias. Statistical analyses were performed using Statistical Product and Service Solutions (SPSS) version 25 (IBM Corp., Armonk, NY).

## Results

Patient and treatment characteristics stratified by primary site are reported in Table 1. Of the 427 total patients identified with grade III SFT/HPC, 180 (42.15%) and 247 (57.85%) had a primary site of the meninges and brain parenchyma, respectively. Patients with grade III SFT/HPC of the meninges were more likely to be older than those with grade III SFT/HPC of the brain parenchyma (*P* = 0.002). The meningeal grade III SFT/HPC group included a higher proportion of patients aged ≥ 70 years compared with the brain grade III SFT/HPC group (17.8% vs. 7.3%), whereas patients aged ≤ 40 years were less frequent in the brain grade III SFT/HPC group (17.8% vs. 23.9%) (P = 0.003). Year of diagnosis, sex, race, household income, primary payer status, facility type, CCI, maximum tumor diameter, treatment utilization (surgery, radiation therapy, or chemotherapy), and times to treatment did not vary by primary tumor site.

**Table 1:** Chi-square analyses of demographics and clinical characteristics of anaplastic hemangiopericytoma by tumor primary site

	Meninges	Brain	Total	*P* value
Total (N, [%])	180 (42.2)	247 (57.8)	427 (100.0)	
Year of diagnosis (N, [%])				
2004–2010	79 (43.9)	119 (48.2)	198 (46.4)	0.380
2011–2017	101 (56.1)	128 (51.8)	229 (53.6)	
Age				
Mean (years, [SE])	55.1 [1.1]	50.8 [0.9]	52.6 [0.7]	**0.002**
$$\le$$ 40 years (N, [%])	32 (17.8)	59 (23.9)	91 (21.3)	**0.003**
40–70 years (N, [%])	116 (64.4)	170 (68.8)	286 (67.0)	
$$\ge$$ 70 years (N, [%])	32 (17.8)	18 (7.3)	50 (11.7)	
Sex (N, [%])				
Male	82 (45.6)	125 (50.6)	207 (48.5)	0.302
Female	98 (54.4)	122 (49.4)	220 (51.5)	
Race (N, [%])				
White	157 (88.7)	214 (87.3)	371 (87.9)	0.609
Black	11 (6.2)	13 (5.3)	24 (5.7)	
Other	9 (5.1)	18 (7.3)	27 (6.4)	
Household income (N, [%])				
< $38,000	24 (14.5)	37 (16.4)	61 (15.6)	0.604
$38,000 – $47,999	37 (22.3)	61 (27.0)	98 (25.0)	
$48,000 – $62,999	42 (25.3)	49 (21.7)	91 (23.2)	
$$\ge$$ $63,000	63 (38.0)	79 (35.0)	142 (36.2)	
Primary payer (N, [%])				
Private insurance & managed care	102 (57.3)	149 (61.1)	251 (59.5)	0.086
Medicaid	16 (9.0)	36 (14.8)	52 (12.3)	
Medicare	49 (27.5)	46 (18.9)	95 (22.5)	
Other	11 (6.2)	13 (5.3)	24 (5.7)	
Facility type (N, [%])				
Community center program	6 (4.0)	6 (3.1)	12 (3.5)	0.053
Comprehensive community cancer program	62 (41.1)	59 (30.7)	121 (35.3)	
Academic/research program	68 (45.0)	115 (59.9)	183 (53.4)	
Integrated network cancer program	15 (9.9)	12 (6.3)	27 (7.9)	
Pacific	18 (11.9)	32 (16.7)	50 (14.6)	
Charlson-Deyo comorbidity index (N, [%])				
0	141 (78.3)	194 (78.5)	335 (78.5)	0.768
1	25 (13.9)	30 (12.1)	55 (12.9)	
$$\ge$$ 2	14 (7.8)	23 (9.3)	37 (8.7)	
Maximum tumor diameter				
Mean (mm, [SE])	48.2 [1.5]	58.2 [4.8]	54.2 [3.0]	0.097
$$<$$ 50 mm (N, [%])	70 (51.5)	90 (45.0)	160 (47.6)	0.244
$$\ge$$ 50 mm (N, [%])	66 (48.5)	110 (55.0)	176 (52.4)	
Treatment class (N, [%])				
Surgery	172 (95.6)	242 (98.0)	414 (97.0)	0.151
Radiation	118 (65.9)	180 (73.2)	298 (70.1)	0.107
Chemotherapy	11 (6.3)	16 (6.8)	27 (6.6)	0.862
Treatment regimen (N, [%])				
No surgery, radiation, or chemotherapy	4 (2.2)	1 (0.4)	5 (1.2)	0.085
Surgery only	54 (30.0)	58 (23.5)	112 (26.2)	0.131
Radiation only	2 (1.1)	2 (0.8)	4 (0.9)	0.750
Chemotherapy only	0 (0.0)	0 (0.0)	0 (0.0)	NR
Surgery, radiation	102 (56.7)	159 (64.4)	261 (61.1)	0.107
Surgery, chemotherapy	1 (0.6)	1 (0.4)	2 (0.5)	0.822
Radiation, chemotherapy	1 (0.6)	1 (0.4)	2 (0.5)	0.822
Surgery, radiation, and chemotherapy	9 (5.0)	14 (5.7)	23 (5.4)	0.763
Treatment times (days, [SE])				
Time from diagnosis to definitive surgery	15.3 [2.6]	11.0 [2.2]	12.8 [1.7]	0.203
Time from definitive surgery to discharge	5.3 [0.4]	5.7 [0.5]	5.5 [0.3]	0.566
Time from diagnosis to radiation initiation	79.4 [6.8]	72.5 [3.9]	75.3 [3.6]	0.344
Duration of radiation	59.1 [13.2]	69.9 [12.3]	65.3 [9.0]	0.555
Time from diagnosis to chemotherapy initiation	59.7 [12.1]	65.0 [9.4]	62.7 [7.3]	0.730
Mortality (N, [%])				
Dead within 30 days of surgery	1 (0.6)	0 (0.0)	1 (0.2)	0.237
Dead within 90 days of surgery	7 (4.1)	2 (0.8)	9 (2.2)	**0.026**
Dead at time of last contact	54 (30.0)	73 (29.6)	127 (29.7)	0.921
Time of last contact				
Mean (years, [SE])	5.2 [0.3]	5.3 [0.2]	5.2 [0.2]	0.664
Median (years)	4.5	4.5	4.5	NR
$$<$$ 1 year (N, [%])	17 (9.4)	18 (7.3)	35 (8.2)	0.782
1–3 years (N, [%])	40 (22.2)	63 (25.5)	103 (24.1)	
3–5 years (N, [%])	42 (23.3)	56 (22.7)	98 (23.0)	
$$\ge$$ 5 years (N, [%])	81 (45.0)	110 (44.5)	191 (44.7)	

**Fig. 1:**
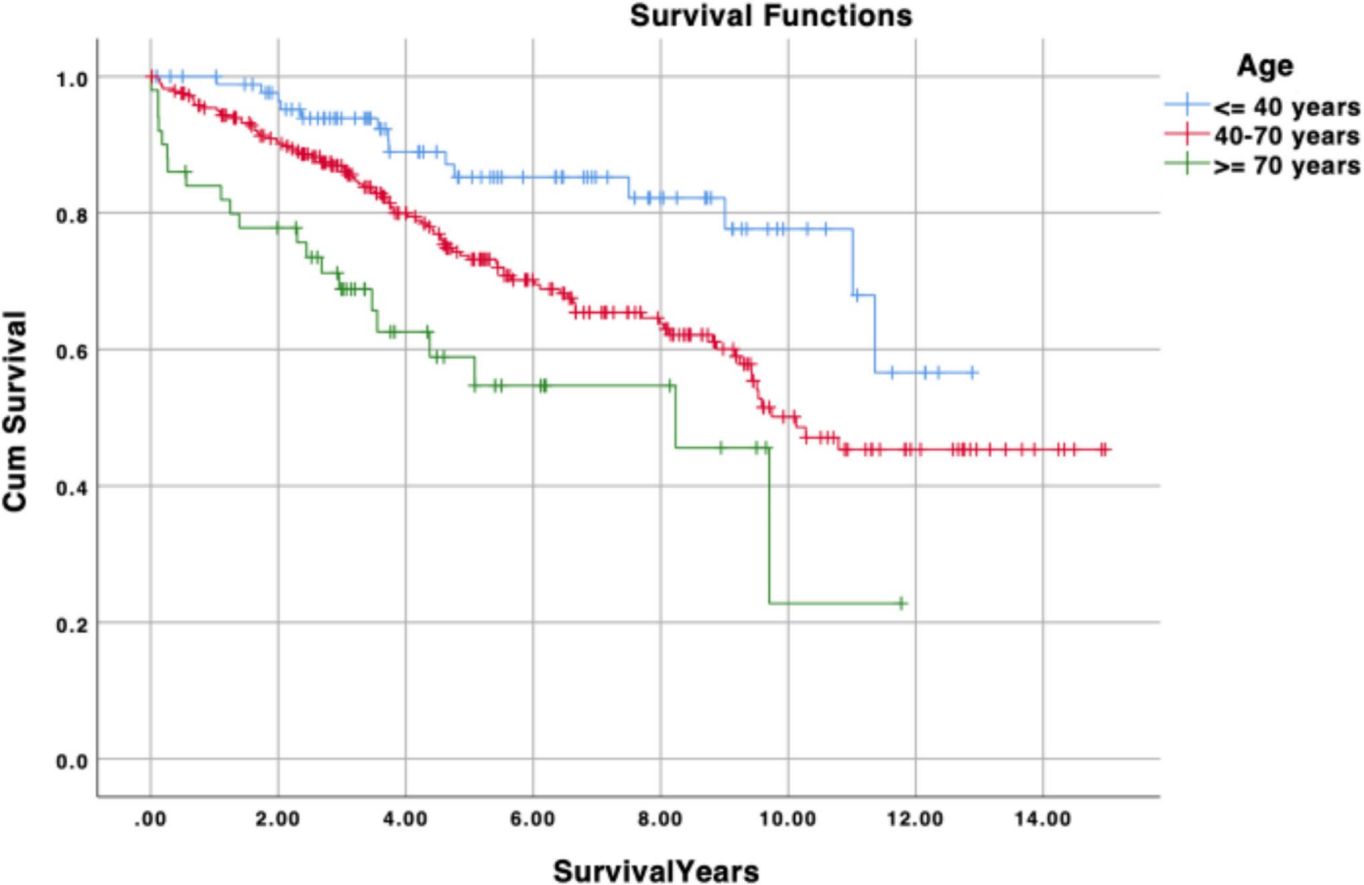
Kaplan–Meier overall survival curves stratified by age group (≤ 40, 40–70, ≥ 70 years) for intracranial grade III SFT/HPC (historically termed AHPC) (log-rank *p* < 0.001). X-axis: time in years. Y-axis: survival probability

Overall survival (OS) of grade III SFT/HPC is summarized in Table 2 and Fig. 1. Mean and median OS were estimated to be 10.0 and 10.8 years, respectively. Mean OS differed significantly by age group, measuring 10.8 years for patients aged ≤ 40 years, 9.9 years for those aged 40–70 years, and 6.6 years for those aged ≥ 70 years (P < 0.001). Patients treated with surgery plus radiation had better OS compared to patients treated with surgery alone, or with a combination of surgery, radiation, & chemotherapy (10.3 vs. 9.4 vs. 5.2 years) (*P* < 0.001). Mean OS did not differ significantly by sex, race, or tumor location.

**Table 2:** Kaplan–Meier log-rank survival analyses of anaplastic hemangiopericytoma by demographics and treatment; NR indicates median not reached

	Mean Survival (years, [SE])	Median Survival (years, [SE])	P value
Overall	10.0 [0.3]	10.8	
Year of diagnosis			
2004–2010	9.9 [0.4]	10.3	0.537
2011–2017	6.6 [0.2]	NR	
Age			
$\le$ 40 years	10.8 [0.5]	NR	** < 0.001**
40–70 years	9.9 [0.4]	10.1	
$\ge$ 70 years	6.6 [0.8]	8.2 [1.6]	
Sex			
Male	9.4 [0.5]	10.1 [0.6]	0.055
Female	10.6 [0.5]	11.4	
Race			
White	9.9 [0.4]	10.3	0.550
Black	8.3 [1.1]	8.1 [1.8]	
Other	10.7 [1.3]	11.0 [3.3]	
Tumor location			
Meninges	10.0 [0.5]	9.7	0.803
Brain	10.1 [0.4]	11.0	
Treatment class			
Surgery	10.1 [0.3]	11.0	**0.046**
No surgery	5.9 [1.2]	9.5 [3.3]	
Radiation	10.0 [0.4]	11.4	0.094
No radiation	9.4 [0.6]	10.3	
Chemotherapy	5.0 [0.6]	4.2 [1.2]	0.009
No chemotherapy	10.1 [0.4]	10.8	
Treatment regimen			
No surgery, radiation, or chemotherapy	2.3 [1.0]	3.7 [0]	** < 0.001**
Surgery only	9.4 [0.7]	9.7 [0.7]	
Surgery, radiation	10.3 [0.4]	11.4	
Surgery, radiation, and chemotherapy	5.2 [0.7]	4.2 [1.2]

Cox proportional hazards regression analysis of overall survival for patients with grade III SFT/HPCis reported in Table 3. Univariable analysis evaluated age, sex, race, household income, primary payer status, facility type, CCI, tumor diameter, tumor location, and treatment regimen. Age, primary payer status, and treatment regimen met the criteria for inclusion in multivariable analysis (*P* < 0.05).

**Table 3:** **Cox** proportional-hazards regression analyses of survival in anaplastic hemangiopericytoma

	Univariate Models		Multivariable Model I		Multivariable Model II	
	HR (95% CI)	*P* value	HR (95% CI)	*P* value	HR (95% CI)	*P* value
Year of diagnosis						
2004–2010	*Ref*					
2011–2017	0.88 (0.59–1.32)	0.537				
Age (years)	1.04 (1.03–1.05)	** < 0.001**	1.03 (1.01–1.05)	**0.006**	1.03 (1.01–1.05)	**0.004**
Sex						
Female	*Ref*					
Male	1.41 (0.99–2.00)	0.056				
Race						
White	*Ref*					
Black	1.06 (0.50–2.28)	0.878				
Other	0.62 (0.25–1.51)	0.288				
Household income						
< $38,000	*Ref*					
$38,000 – $47,999	0.95 (0.56–1.63)	0.854				
$48,000 – $62,999	0.72 (0.41–1.27)	0.258				
$$\ge$$ $63,000	0.65 (0.38–1.10)	0.109				
Primary payer						
Medicare	*Ref*		*Ref*		*Ref*	
Private insurance & managed care	0.32 (0.212–0.47)	** < 0.001**	0.55 (0.33–0.91)	**0.021**	0.57 (0.39–0.96)	**0.033**
Medicaid	0.61 (0.34–1.09	0.097	1.15 (0.56–2.36)	0.697	1.26 (0.60–2.60)	0.547
Other	0.68 (0.32–1.44)	0.681	1.23 (0.50–3.04)	0.653	1.23 (0.50–3.05)	0.660
Facility type (N, [%])						
Community center program	*Ref*					
Comprehensive community cancer program	4.20 (0.58–30.47)	0.156				
Academic/research program	4.18 (0.58–30.15)	0.156				
Integrated network cancer program	5.48 (0.70–42.87)	0.105				
Charlson-Deyo comorbidity index						
0	*Ref*					
1	1.23 (0.73–2.06)	0.440				
$$\ge$$ 2	1.50 (0.85–2.62)	0.160				
Maximum tumor diameter (mm)	1.00 (0.99–1.00)	0.676				
Tumor location						
Meninges	*Ref*					
Brain	0.96 (0.67–1.36)	0.803				
Treatment class						
Surgery	0.44 (0.20–1.01)	0.052	0.58 (0.23–1.45)	0.244		
Radiation	0.73 (0.51–1.06)	0.095	0.80 (0.54–1.18)	0.253		
Chemotherapy	2.36 (1.22–4.57)	**0.011**	2.37 (1.20–4.66)	**0.013**		
Treatment regimen						
No surgery, radiation, or chemotherapy	*Ref*				*Ref*	
Surgery only	0.14 (0.04–0.47)	**0.001**			0.16 (0.05–0.55)	**0.003**
Surgery, radiation	0.10 (0.03–0.32)	** < 0.001**			0.14 (0.04–0.46)	**0.001**
Surgery, radiation, and chemotherapy	0.25 (0.07–0.95)	**0.042**			0.36 (0.09–1.39)	0.138

Multivariable Model I includes treatment modalities as independent binary variables: surgery (yes/no), radiation (yes/no), and chemotherapy (yes/no). This model estimates the independent effect of each treatment while adjusting for collinearity. Multivariable Model II includes mutually exclusive treatment regimen categories: no treatment (reference), surgery only, surgery + radiation, and surgery + radiation + chemotherapy. This model estimates the effect of each treatment combination on survival relative to no treatment.

Abbreviations: *HR* Hazard ratio, *CI* Confidence interval, *Ref* Reference group

On multivariable analysis, increasing age (Hazards Ratio [HR] 1.03, 95% confidence intervals [CI] 1.01–1.05) was associated with a higher mortality risk. Private insurance, compared with Medicare, was associated with improved survival (HR 0.57, 95% CI 0.39–0.96). Relative to no treatment, both surgery alone (HR 0.16, 95% CI 0.05–0.55) and surgery combined with radiation therapy (HR 0.14, 95% 0.04–0.46) were independently associated with improved overall survival (all *P* < 0.05).

## Discussion

In this national analysis of 427 patients with intracranial grade III SFT/HPC, we provide contemporary real-world evidence on treatment patterns and prognostic factors for this rare tumor. We observed an overall median survival of approximately 10.8 years from diagnosis, although survival varied widely by age. Patients under 40 years of age had notably prolonged survival, with over 70% surviving 10 years or more, whereas those over 70 faced a much poorer outlook (median survival ~ 6–8 years). This age effect is consistent with prior studies showing age at diagnosis to be a key prognostic factor in grade III SFT/HPC [7, 26]. For instance, the population-based SEER study by Kinslow et al. [7] reported a median survival at 155 months (~ 12.9 years) across all grades of CNS SFT/HPC, and found that older age increased the risk of death by ~ 3.8% per year (HR 1.038 per year), which aligns closely with our findings (HR ~ 1.03). Our focus on grade III tumors likely explains the slightly shorter median OS we found, as lower-grade SFTs have a more indolent course. Nevertheless, our results affirm that advanced age is associated with worse prognosis in these tumors, likely reflecting both decreased physiological reserve and potentially more aggressive disease biology in older patients [26]. 

A primary aim of our study was to examine the impact of treatment modalities on survival. We found that patients who underwent surgical resection, particularly when combined with adjuvant radiotherapy, had significantly improved survival outcome. This echoes the prevailing consensus and prior research that maximal safe resection followed by radiotherapy offers the best chance of durable local control for high-grade SFT/HPC [20, 23]. In our cohort, surgery plus radiation reduced the hazard of mortality by ~ 86% compared to no treatment in multivariable analysis, and had superior unadjusted 5- to 10-year survival compared to surgery alone. Although surgery and radiation did not show independent significance in one multivariable model (likely due to collinearity and limited sample of non-surgical cases), the benefit of combined modality therapy is strongly supported by our regimen-based analysis. This finding is in line with multiple prior studies. Sonabend et al. (2014) examined 227 patients with CNS HPC from SEER and found gross total resection (GTR) combined with adjuvant radiotherapy conferred a significant overall survival benefit (HR 0.31) that was not observed with GTR alone [23]. Similarly, Kinslow et al. (2018) noted that, among malignant and borderline SFT/HPC cases, GTR + RT significantly improved survival compared to GTR alone [7]. Our NCDB analysis reinforces these findings in a more recent cohort: patients receiving adjuvant radiotherapy after resection had longer median survival than those with surgery alone (11.4 years vs 9.7 years), supporting the recommendation of postoperative radiotherapy for grade III SFT/HPC. The likely benefit of radiation is in controlling microscopic residual disease and reducing recurrence, which may translate into improved long-term survival [7]. It should be noted that one NCDB study by Trifiletti et al. (2017) did not find a significant impact of radiotherapy on OS in CNS HPC; however, that study included a mix of grades (and possibly more grade II tumors) and observed that radiation was mainly beneficial for local control [26]. Our data, focused on grade III, align more with the recent evidence that adjuvant radiotherapy is particularly valuable in high-risk patients. [8] Indeed, a 2023 multi-dataset analysis by Kinslow et al. introduced a risk stratification model and found that radiotherapy was associated with improved survival in grade III (high-risk) SFT/HPC patients, whereas it had less impact in lower-grade groups. [8] This stratified benefit corresponds with our observation that the surgery + RT combination yielded the best outcomes in the high-grade population.

Our study also highlights the importance of gross total resection whenever feasible. While NCDB lacks direct coding for extent of resection, the strong survival advantage in surgically treated patients suggests that achieving resection, especially complete removal, is critical. Prior institutional series have demonstrated that incomplete resection is associated with higher recurrence and mortality. [23] Stessin et al. (2013), in a SEER analysis of 76 meningeal HPC cases, noted that postoperative radiotherapy significantly improved cause-specific survival, particularly in patients with subtotal resection (HR 0.09) [23]. This emphasizes that even if GTR is not attainable, adjuvant radiation can partly compensate by controlling residual disease. In our cohort, it is reasonable to assume most patients had resection; those who did not (or had only a biopsy) would have had worse outcomes. Thus, our findings support aggressive surgical management as a cornerstone of therapy, followed by radiotherapy to address any residual tumor cells and mitigate the high recurrence propensity of grade III SFT/HPC.

In contrast to surgery and radiation, chemotherapy was not associated with improved survival in our analysis. In fact, receipt of chemotherapy was linked to significantly worse outcomes (HR 2.37 for death). We interpret this not as a direct harmful effect of chemotherapy, but as an indication of selection bias: chemotherapy tends to be given to patients with more advanced or refractory disease (for example, those with metastases or multiple recurrences). Since the NCDB does not record disease recurrence or metastasis at presentation in a granular way, chemotherapy use likely served as a surrogate for aggressive tumor behavior. Our results corroborate those of Wang et al. (2020), who analyzed 1243 intracranial HPC cases from SEER and also found that adding chemotherapy did not improve outcomes (worsened patient survival, p < 0.001) [27]. Similarly, Trifiletti et al. observed chemotherapy was associated with inferior OS (HR = 2.66) in their NCDB cohort [26]. To date, there is no established effective chemotherapy regimen for SFT/HPC, and evidence is limited to small case series. Agents such as doxorubicin (Adriamycin), ifosfamide, temozolomide, and anti-angiogenic drugs like bevacizumab have been tried, given the tumor’s sarcoma-like and vascular nature, but responses are variable and often transient [12, 13, 17, 21, 22]. Chemotherapy was associated with worse OS in this dataset; however, this finding most likely reflects confounding by indication (chemotherapy preferentially used in higher-risk clinical scenarios) and should not be interpreted as evidence of direct treatment harm. The worse survival of chemo-treated patients likely reflects the underlying disease severity; we have explicitly acknowledged this selection bias in our interpretation and in the abstract’s conclusion to avoid misguiding readers. Importantly, our study design could not correct for immortal time bias: patients must survive long enough post-surgery to receive radiotherapy or chemotherapy, which can exaggerate the apparent benefit of treatments like radiotherapy in retrospective data. This limitation may overestimate the apparent benefit for radiotherapy because patients must survive long enough after diagnosis/surgery to receive radiotherapy. The NCDB does not capture chemotherapy intent, line of therapy, regimen, or recurrence status, limiting interpretation of systemic therapy associations. We did not perform a landmark analysis or time-dependent covariate analysis for radiotherapy, which is a limitation. Nonetheless, the consistency of our results with other large studies lends credibility to the observed associations.

One intriguing observation was the independent association of primary payer (insurance) with survival. Patients with private insurance or managed care plans had significantly better survival than those on Medicare (the reference group in our model), even after adjusting for age, which is a major determinant of insurance. This could imply that socioeconomic factors and access to care influence outcomes in rare CNS tumours. Privately insured (often non-elderly) patients may have more timely access to specialized neuro-oncologic care, second opinions, or advanced therapies, whereas Medicare patients, who are mostly ≥ 65, might have had more comorbidities or less aggressive treatment (though we adjusted for comorbidity index). It is also possible that this finding partly reflects residual confounding by age, since Medicare includes virtually all patients over 65, who did worse overall. However, even with the multivariable model accounting for age as a continuous variable, insurance emerged still as a significant factor. Similar disparities have been noted in other cancers where uninsured or publicly insured patients have worse outcomes, often attributed to later presentation or differences in treatment patterns [29]. In SFT/HPC specifically, this is a novel insight, as prior database studies have not commented on insurance status. Our result should prompt further investigation into healthcare delivery factors for rare tumor management. Ensuring that all patients, regardless of age or insurance, have access to optimal surgical and radiotherapy expertise is vital.

Tumor location (meninges vs. brain) was not found to affect survival in our analysis, once treatment and age were considered. This is an important point because intracranial SFT/HPCs are thought to originate from meningeal pericytes; a “brain” designation likely means a tumor arising in the dura that has invaded or is situated adjacent to brain tissue, rather than a truly distinct entity. The lack of survival difference supports that anaplastic SFT/HPC behaves similarly regardless of exact intracranial location, with outcomes driven more by resectability and biology than site of origin. This observation addresses a potential coding issue in big databases: some cases coded as “brain” could simply be dural tumors coded under brain subsites. As CNS HPCs originate from the meninges, those coded as “brain” may reflect more aggressive behavior (invasion into brain parenchyma) rather than a different cell of origin. However, because SFT/HPC is typically dural-based, “brain” coding in registry data may reflect anatomic coding practices rather than a biologically distinct site of origin. Our data did show meningeal cases tended to exist in older patients, but importantly we did not find a poorer prognosis for brain-invading tumors per se. Prior series have suggested that tumors with brain invasion may recur earlier, but the primary determinant of outcomes was extent of resection and grade, not location alone [23]. Thus, clinicians should approach all intracranial SFT/HPCs with aggressive management, regardless of whether they are attached to dura or located intraparenchymally, as the underlying pathology and treatment principles are the same.

## Limitations

This study’s retrospective nature and use of the NCDB impose several limitations. First, as with all registry analyses, there is potential for miscoding and misclassification, as has been discussed in detail in several papers [5, 15]. The definition of “anaplastic hemangiopericytoma” evolved over our study period (2004–2017) due to changes in WHO classification. In addition, because NCDB coding is not centrally adjudicated, both histology and grade may vary by institution and era, particularly across the WHO 2016 and WHO 2021 classification changes, introducing potential time-related coding drift. It is possible that some lower-grade SFTs were initially mislabeled as grade III SFT/HPC or vice versa, especially earlier in the timeframe. We attempted to mitigate this by using strict ICD-O coding and limiting analysis to cases designated as malignant hemangiopericytoma by pathologists at the time of diagnosis. Still, the NCDB would have classified each case according to the ICD-O histology noted; any shifts in diagnostic criteria over time could affect the consistency of our cohort [4]. Second, the NCDB lacks detailed clinical information on several prognostically important variables. We do not have information on performance status or neurological function of patients, which influence treatment decisions and survival. We also lack genetic and molecular data: for example, NAB2-STAT6 fusion status (though nearly universal in SFT/HPC), or other mutations that might modulate prognosis. Histopathological details such as Ki-67 index, extent of necrosis, or specific mitotic counts, which could further risk-stratify grade III tumors, are not recorded. Additionally, as noted, we do not know the extent of surgical resection (GTR vs. partial resection), nor the dose and technique of radiotherapy (e.g. external beam vs. stereotactic radiosurgery) or specifics of chemotherapy regimens administered. The absence of these data means we must infer some effects (for example, we attribute better survival to likely gross total resections, but cannot confirm how many had GTR). The NCDB also does not capture recurrence or metastasis occurrence during follow-up, so we could not directly analyze progression-free survival or patterns of failure. We also could not account for distant metastases as presentation; some patients might have had extracranial metastatic disease (NCDB has a field for metastasis but completeness is variable), which would heavily influence the decision to administer chemotherapy and the survival outcome. Omission of metastatic status in our model is a limitation that could confound the chemotherapy-survival association (patients with metastases likely received chemotherapy and had worse survival). Third, our survival analysis may suffer from immortal time bias with respect to radiotherapy, as discussed: patients must survive the post-operative period to receive radiation, which can artificially inflate survival for the radiotherapy group in retrospective data. While the consistency of benefit seen in other studies suggests a true effect, caution is warranted in interpreting the magnitude of the radiotherapy benefit from our study alone. Lastly, the NCDB represents outcomes from the United States only; thus, our findings might not be generalizable to regions with different healthcare systems or genetic backgrounds. Given that SFT/HPC is a rare tumor with potentially varying incidence across ethnicities, [19] international data would be valuable. A tumor so influenced by gene fusions might have biological variations across populations, which means that multinational studies are needed to validate prognostic factors.

Despite these limitations, our study has notable strengths. It is, to our knowledge, the largest series focusing on intracranial grade III SFT/HPC reported to date, leveraging a robust hospital-based registry. By restricting to grade III, we provide more homogeneous insight into the malignant end of the SFT spectrum, in contrast to prior studies that combined all grades. We performed a comprehensive multivariable analysis that identified predictors, something few earlier studies on AHPC have done. In fact, our study appears to be the only one on AHPC that includes a multivariable Cox analysis using a large national sample. We found that, even after adjustment, the effects of age, treatment, and insurance remained significant, highlighting their importance. Additionally, our dataset spans 14 years, capturing modern treatment era practices. We also compared our results with those from other key studies (including recent 2022–2023 analyses), thereby placing our findings in the evolving context of SFT/HPC management. Ultimately, our study provides real-world evidence that surgery and radiotherapy are crucial for improving survival in grade III SFT/HPC, whereas systemic therapy has yet to show benefit: information that can guide clinicians and patients in decision-making. Moreover, the observed survival rates offer a hopeful perspective that long-term remission is achievable in a significant subset of patients, especially when optimal care is delivered.

## Conclusions

In this NCDB cohort of 427 patients with intracranial grade III SFT/HPC, median overall survival was 10.8 years. Older age was linked to worse survival. Patients with private insurance and those treated with surgery plus radiotherapy had the best outcomes, while chemotherapy showed no survival benefit. These findings support aggressive surgical management with adjuvant radiotherapy as the cornerstone of care for grade III SFT; however, continued advances in therapeutic strategies are needed to further improve survival in this rare and clinically devastating disease.

## Data Availability

The data that support the findings of this study are available from the National Cancer Database (NCDB) Participant User File program. Restrictions apply to the availability of these data under the NCDB Data Use Agreement; therefore, the data are not publicly available. Data may be obtained through application to the American College of Surgeons Commission on Cancer.
